# Keeping the beat against time: Mitochondrial fitness in the aging heart

**DOI:** 10.3389/fragi.2022.951417

**Published:** 2022-07-26

**Authors:** Arielys Mendoza, Jason Karch

**Affiliations:** ^1^ Department of Integrative Physiology, Baylor College of Medicine, Houston, TX, United States; ^2^ Cardiovascular Research Institute, Baylor College of Medicine, Houston, TX, United States

**Keywords:** mitochondria, cell death, mitophagy, autophagy, ROS, metabolism, mitochondrial biogenesis, mitochondrial fitness

## Abstract

The process of aging strongly correlates with maladaptive architectural, mechanical, and biochemical alterations that contribute to the decline in cardiac function. Consequently, aging is a major risk factor for the development of heart disease, the leading cause of death in the developed world. In this review, we will summarize the classic and recently uncovered pathological changes within the aged heart with an emphasis on the mitochondria. Specifically, we describe the metabolic changes that occur in the aging heart as well as the loss of mitochondrial fitness and function and how these factors contribute to the decline in cardiomyocyte number. In addition, we highlight recent pharmacological, genetic, or behavioral therapeutic intervention advancements that may alleviate age-related cardiac decline.

## 1 Introduction

Extending life expectancy while attenuating the negative effects of aging is, arguably, the overall goal of health sciences (Sierra et al., 2009). Reducing the maladaptive effects of aging not only lengthens survival, but also preserves bodily functions and enhances overall fitness into the later stages of life. (Vaupel, 2010; Olshansky, 2018). Since the 19th century we have successfully prolonged the average human lifespan to a natural maximum of 115 years, with the likelihood of individuals surviving an age greater than 125 years being less than 1 in 10,000 (Dong et al., 2016). This increase in lifespan is due to the major contributions of modern medical advancements which include preventative measures such as immunizations and the administration of symptom-targeting therapies like insulin (Ben-Haim et al., 2017). Inevitably, as the population’s life expectancy increased nearly two-fold within the last century, systematic conditions such as cancer and heart disease now dominates the majority of late-age mortalities (Crimmins, 2015). Undoubtedly, understanding age-related changes that occur in the heart over time in order to therapeutically counter their effects is crucial for preventing heart disease.

Cardiovascular disease (CVD) includes a collection of pathological heart disorders such as heart failure (HF), ischemia-reperfusion (I/R) injury, atherosclerosis, and arrhythmias, which together culminate into the majority of deaths in developed countries (Tang X. et al., 2020). Cardiomyocyte viability is a major contributing or initiating factor for many forms of CVD, and mitochondrial health and function are critical to myocyte contractility and survival. In this review, we will examine the major findings regarding the time-dependent decline of the heart with respect to: 1) cardiomyocyte drop-out, 2) maladaptive shifts in major signaling pathways, 3) the contributions of reactive oxygen species (ROS) and 4) mitochondrial dysfunction. Finally, we will highlight the current efforts to preserve heart health and the novel developments in medical interventions, exercise, and diet to delay the negative consequences of cardiac aging.

## 2 Cardiomyocyte drop-out over time

**FIGURE 1:**
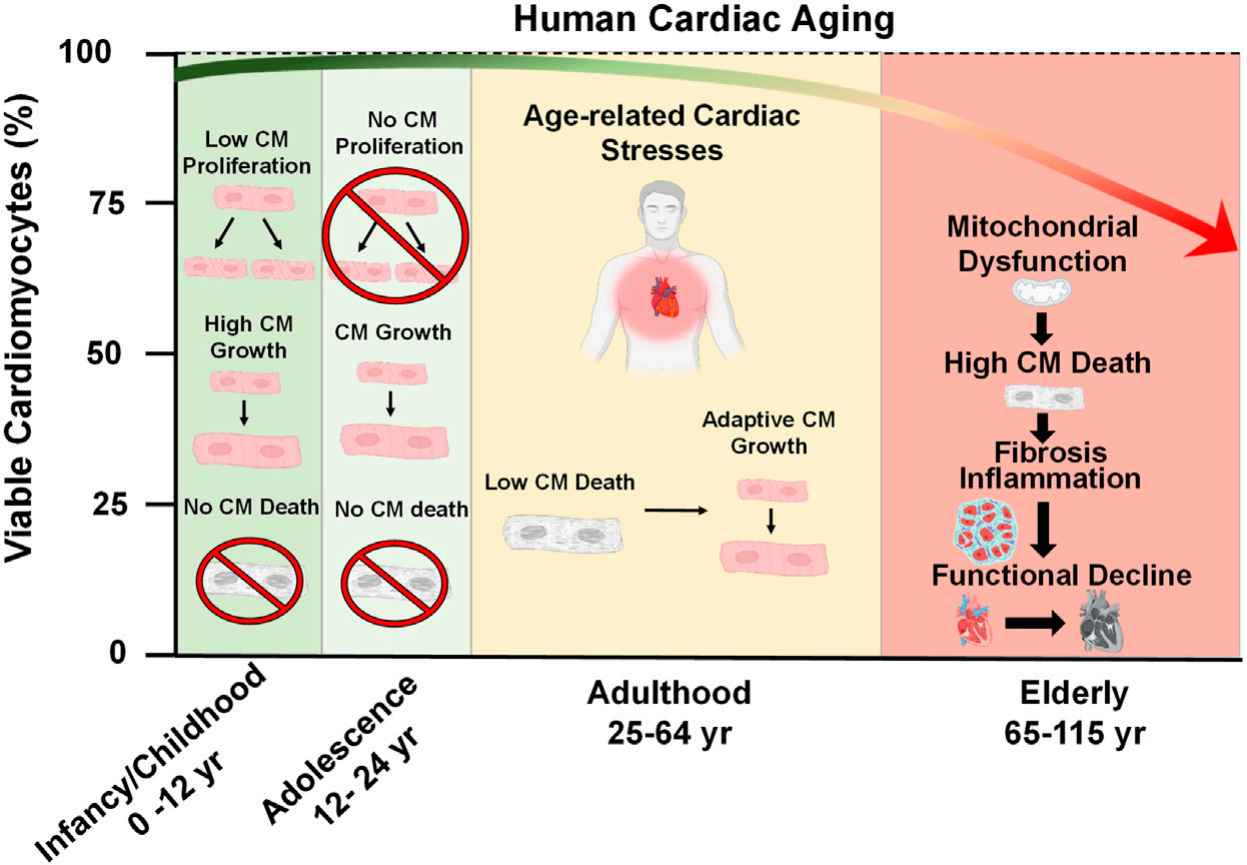
Cardiac Aging. At the time of birth, the postnatal human heart exhibits a brief period of cell growth through proliferation. However, this proliferative capacity is lost shortly after birth and heart growth proceeds by an increase in cardiomyocyte (CM) size throughout adolescence (ages 12–24). During adulthood (ages 25–64), a decline in heart function occurs due to the emergence of pathological age-related stresses, such as imbalances in force distribution per myocyte as a result of natural CM dropout, altered metabolism, decreased ATP generation, and accumulation of reactive oxygen species. These stresses consequently lead to mitochondrial dysfunction, increased CM death, inflammation, fibrosis, and the overall functional decline of the heart late in life.

During embryonic development, cardiac progenitor cells from the mesoderm arise and differentiate into their cardiomyocyte fates, which then form the cardiac tube and continue to populate what will become the chambers of the primitive atria and ventricles (Asp et al., 2019). Shortly after birth, the mammalian heart loses its regenerative capacity (Figure 1) (Foley and Mercola, 2004). Unlike the embryonic heart, the adolescent heart loses its ability to proliferate and instead undergoes growth due to an increase in cell size (Figure 1) (Günthel et al., 2018). This arrest of proliferative capacity experienced by cardiomyocytes leads to a total withdrawal from the cell cycle, induced by the downregulation of pro-mitotic factors such as cyclins and cyclin-dependent kinases (Hesse et al., 2018). In contrast, cyclin inhibitors such as p21, p27, and p57 which antagonize cardiomyocyte mitosis also become upregulated upon birth (Tane et al., 2014; Hesse et al., 2018). Therefore, adult cardiomyocytes are post-mitotic and terminally differentiated (Bergmann, 2019). Additionally, the adult heart has an extremely low potential for regeneration (Rizzo et al., 2018). There is evidence for low level regeneration that occurs in the adult human heart which ranges between a rate of 0.5–2% per year, however, this endogenous regenerative capacity is not sufficient to overcome a catastrophic injury such as a myocardial infarction (Senyo et al., 2013; Bergmann et al., 2015). Earlier reports have claimed the existence of cardiac progenitor pools in extra cardiac (i.e. bone marrow) or resident tissues that can give rise to new cardiomyocytes (Beltrami et al., 2003; Martin et al., 2004; Matsuura et al., 2004; Oyama et al., 2007). Resident cardiac stem cells were identified by their expression of hematopoietic stem cell markers such as c-kit and stem cell antigen 1 (Beltrami et al., 2003). However, the majority of these claims have been discredited and retracted, causing much controversy within the field (Li H. et al., 2020). The classification of cardiac stem cells based on stem cell factor expression alone was challenged (Sultana et al., 2015; Vicinanza et al., 2017), and lineage-tracing techniques demonstrated that previously described cardiac resident stem cells do not produce cardiomyocytes (Van Berlo et al., 2014; He et al., 2017). A recent consensus statement was published to redefine the narrative on cardiomyocyte renewal and to assert agreement of the low regenerative potential of adult myocardium at both homeostatic and post-injury conditions (Figure 1) (Eschenhagen et al., 2017). Therefore, cardiomyocyte viability must stand the test of time, as the cardiomyocytes present at young adulthood are the same cardiomyocytes present later in life.

Since cardiomyocytes are naturally long-lived cells it is logical to assume that they are naturally resistant to cell death. Indeed, many cell death-regulating factors are suppressed at the transcript level compared to other tissues in the body (Patel and Karch, 2020). However, cardiomyocytes are not invincible and throughout life, there is a slow but gradual loss of cardiomyocytes, referred to as cardiomyocyte drop-out. It has been estimated that roughly 33% of cardiomyocytes of the left ventricle dropout naturally during a person’s lifetime, according to a study which reported that between the ages of 17–90 years, the human heart experiences a drop-out rate of 38 million cardiomyocytes/yr within the left ventricle and a cell volume enlargement of the surviving myocytes of 110 μm^3^/yr (Figure 1) (Olivetti et al., 1991). As a highly contractile organ, the heart must sustain appropriate levels of rigidity and elasticity to maintain shape, distribute force, and efficiently eject blood in a controlled fashion (Vikhorev and Vikhoreva, 2018). The slow decline in myocytes, which are the contractile unit of the heart, is responsible for increasing mechanical stress per myocyte (Bernhard and Laufer, 2008). Once a sufficient amount of dropout has occurred, the heart may compensate by becoming hypertrophic in order to maintain the workload (Lakatta, 2000; Fajemiroye et al., 2018). In addition to myocyte hypertrophy, activated fibroblasts (myofibroblasts) will proliferate and infiltrate the heart to fill the residual space left by dead CMs in a process known as fibrosis (Gourdie et al., 2016). A recent study evaluated the physical fitness of 104 healthy volunteers ranging from 20 to 76 years of age and demonstrated that fibrosis negatively impacts the exercise fitness of older individuals, as their hearts cannot eject an adequate volume of blood to the body (Pandey et al., 2020).

**FIGURE 2:**
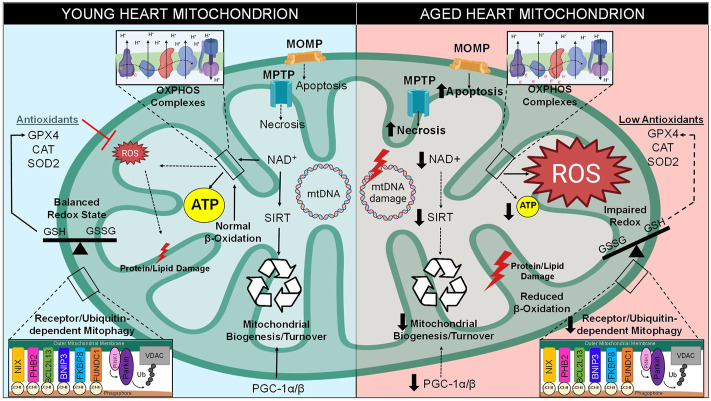
Mitochondrial Dysfunction in Aging. There are various intracellular pathways that become altered over time, especially with regard to mitochondrial function. Between the stages of young and late adulthood, mitochondria within the cardiomyocytes experience a decreased level of biogenesis due to decreased expression of PGC-1 and downregulation of NAD^+^-dependent sirtuins. A metabolic shift occurs in old hearts, resulting in greater glucose and less fatty acids utilization for energy production. Mitochondrial quality control by mitophagy induction is also compromised. Cell death levels increase in the form of apoptosis and necrosis, which utilize mitochondrial outer membrane permeabilization (MOMP) through the Bcl-2 family and mitochondrial permeability transition pore (MPTP), respectively. Excess ROS is generated through increase electron transport chain (ETC) leak, resulting in increased levels of mitochondrial DNA (mtDNA) damage, imbalance of CM redox states, damage to lipids/proteins within the mitochondria, and the inability of antioxidants to clear ROS from the microenvironment. Together, these stressors lead to a decrease in mitochondrial function and lower levels of ATP production.

Furthermore, the most common cause of heart failure is due to a sudden loss of a large portion of cardiomyocytes due to myocardial infarction (MI) (Hofstra et al., 2000). This occurs when arteries that provide oxygenated blood to the heart become blocked, which commonly requires a lifetime of plaque build-up to occur. Preserving cardiomyocyte viability in the face of these extreme events will limit the onset of age-related cardiomyopathies, and a better understanding of what pathways are engaged during cardiomyocyte drop-out may provide insight on how to fortify myocytes against a life time of stressors. The majority of cardiovascular diseases involve or are initiated by the irreplaceable death of cardiomyocytes (Benjamin et al., 2019). In terms of cardiac injury and disease states, at least six types of cell death have been previously described (Patel and Karch, 2020). Of these cell death mechanisms, mainly two forms of cell death, apoptosis and necrosis, occur in the aging heart (Figure 2) (Kajstura et al., 1996). Apoptosis is regulated mainly by the B-cell lymphoma 2 (BCL-2) family at the level of the mitochondria, where pro-apoptotic family member effectors, BAK and BAX, can form homo/hetero-oligomeric pores on the outer mitochondrial membrane (OMM) to induce mitochondrial outer membrane permeability (MOMP). MOMP is prevented by anti-apoptotic BCL-2 family members such as BCL-2. BCL-xL, MCL-1, which directly interact with pro-apoptotic BCL-2 family members to inhibit BAX/BAK oligomerization (Otera et al., 2013). Once MOMP occurs, cytochrome c is released into the cytosol which leads to the formation of the apoptosome and the initiation of the caspase cascade (Garrido et al., 2006). Activation of execution caspases 3 and 7 (CASP3, CASP7) result in proteolysis, morphological membrane blebbing, and cell shrinkage, which are all considered hallmarks of apoptosis (Tait and Green, 2010).

A recent study determined that intrinsic apoptosis signaling becomes altered in the cardiac muscles of old Fischer 344 rats (20 months), such that pro-apoptotic Bax protein is increased by at least 69% and anti-apoptotic protein levels of Bcl-2 were reduced by 70% comparative to very young rat hearts (1 month) (No et al., 2020a). This group also observed a higher number of cleaved CASP3 and TUNEL-positive myocytes within elderly rat hearts as well, which may indicate that the change in expression of apoptotic regulators may exacerbate aging-induced cardiac dysfunction. One team of investigators concluded that subjecting senile rats to 12 weeks of exercise helped attenuate age-induced apoptosis, cardiac remodeling, and BAX/BCL-2 ratio in the heart (Kwak et al., 2006). Currently, there lacks consensus on the contribution of apoptosis to age-related cardiomyocyte drop-out (Li H. et al., 2020). Early studies on elderly human patients determined that levels of apoptotic death increase with age in the myocardium (James, 1994), however, more recent investigations also using human subjects failed to detect a correlation between this form of age-related cardiomyocyte drop-out and apoptosis (Mallat et al., 2001).

Necrotic cell death has also been implicated in cardiac aging. In contrast to apoptosis, the necrotic death of the cardiomyocyte results in the release of intracellular components due to plasma membrane permeabilization, which causes an inflammatory response upon the neighboring myocardial tissue (Bernhard and Laufer, 2008). One investigation determined that the inhibition of regulated necrosis by administration of necrostatin-1, which specifically inhibits of necroptosis (a sub-type of necrosis), can significantly reduce infarct size by 37% of I/R injury-induced aged rat hearts compared to controls (Garvin et al., 2018). Necroptosis is governed by the receptor-interacting protein kinases 1 and 3 (RIPK1, RIPK3) which together become the necrosome which phosphorylates mixed lineage kinase domain-like protein (MLKL) which, upon activation, induces detrimental membrane permeabilization and subsequent death via necroptosis (Sun et al., 2012). In a recent investigation, an anti-aging drug known as Metformin was administered to aged WT mice for 4 weeks prior to being subjected to I/R injury which resulted in a decrease in infarct size compared to aged hearts that received the vehicle control (Li C. et al., 2020). This study determined that Metformin could be targeting the necroptosis pathway in a RIP1-RIP3-dependent mechanism, thus providing anti-aging properties to injured and aged myocardium (Li C. et al., 2020). In addition, the same group observed that RIPK3-deficient (Ripk3^−/−^) murine hearts became more resistant to I/R-induced myocardial necrosis comparative to WT counterparts. While the association of necroptosis to age-related cardiomyocyte mortality is still relatively new in the literature, these reports certainly support the hypothesis of this form of regulated necrosis occurring in the old heart.

In addition to maturing cardiac tissue becoming increasingly impaired due to cardiomyocyte drop-out, altered calcium (Ca^2+^) handling has also been associated with the decline of cardiomyocyte function. Physiologically, Ca^2+^ is central to various signaling pathways and is tightly regulated by influx/efflux channels and pumps (Modesti et al., 2021). It is well understood, that the dysregulation of Ca^2+^ within the confines of the cell can lead to the overloading of the ion to toxic degrees. The mitochondrial calcium uniporter (MCU) is a known transporter of Ca^2+^ into the mitochondrial matrix, which when exceeds physiological limits, may induce maladaptive responses such as the opening of the mitochondrial permeability transition pore (MPTP). The opening of the MPTP, a non-specific voltage-dependent pore, results in a loss of mitochondrial membrane potential, a reduction in adenosine triphosphate (ATP) generation, and overall mitochondrial dysfunction if prolonged opening occurs (Halestrap and Pasdois, 2009). Although the molecular identity of the multi-protein pore has yet to be fully elucidated, its known regulators are the adenine nucleotide translocases (ANTs) and peptidyl-prolyl isomerase cyclophilin D (CypD) (Karch and Molkentin, 2014). One previous study reported that deacetylation at lysine 166 of CypD can suppress mitochondrial dysfunction and age-associated cardiac hypertrophy (Hafner et al., 2010).

There is a consensus that an overload of Ca^2+^ perturbs aged cardiac tissue, evidenced by several reports (Hunter et al., 2012; No et al., 2018) Furthermore, aged myofibrils have been previously shown to grow insensitively to intracellular Ca^2+^ concentrations, which is thought to reflect a lack of contractility and prolonged relaxation in older individuals (Bernhard and Laufer, 2008). Accurately measuring the extent of aging-dependent cardiomyocyte dropout *in vivo* is extremely challenging and will require novel methods in order to investigate the potential cell death mechanisms that are utilized. Once these methods are established, genetic or pharmacological inhibition of individual cell death pathways will reveal which mechanisms contribute to age-related cardiomyocyte drop out.

## 3 Decline in mitochondrial ATP generation

One organelle at the center of cell death and aging is the mitochondrion. The mitochondrial theory of aging postulates that the accumulation of damaged, genetically mutated, respiratory dysfunctional, and excessively ROS-producing mitochondria over time correlates strongly with age-related heart disease (Kowald, 2001; Loeb et al., 2005). Thus, there is a potential relationship between mitochondrial fitness and cardiomyocyte aging. There are two main types of mitochondria within cardiomyocytes, one of which are located beneath the sarcolemma (subsarcolemmal mitochondria, SSM) or between myofribrils (interfibrillar mitochondria, IFM) (Palmer et al., 1977). Interestingly, there is a decreased number of IFM in elderly rat hearts (24–28 months) but no change in SSM content comparative to younger adult hearts (6 months old), which indicates that there is a potential change in ATP levels within micro domains of the aged myocyte (Fannin et al., 1999). Structurally, there are no apparent differences between mitochondria of adult and aged rat hearts using transmission electron microscopy (Palmer et al., 1985; Lesnefsky et al., 2016). However, other groups have reported a noticeable difference in the appearance of mitochondrial cristae between adult and elderly myocyte mitochondria, as electron microscopy revealed that SSM isolated from 24-month old rats have normal lamelliform cristae, whereas IFM cristae display a more tubular phenotype (Riva et al., 2005). Another study identified an age-related decrease in the surface area of the inner mitochondrial membrane (IMM) between 3-months and 24-month-old (El’darov et al., 2015).

As the heart continuously works to supply blood throughout the body, it must rely on mitochondria to generate a sufficient amount of ATP to satisfy the high energetic demand of the cardiomyocyte, which makes mitochondria a major contributing factor to cardiac aging (Tang X. et al., 2020). Due to this absolute necessity for energetic substrate, mitochondria form dense linear networks along sarcomeres, occupying at least 30–40% of cardiomyocyte cell volume (Cao and Zheng, 2019). Within the developing fetal and immediate postnatal heart, glucose is the main source for energy production whereas the utilization of fatty acids for substrate at this stage of life is very minimal due to low circulation of fats and high availability of lactate (Girard et al., 1992; Lopaschuk and Jaswal, 2010). However upon days after birth, the mammalian heart no longer relies on anaerobic glycolysis for its main form of energy transduction (Piquereau and Ventura-Clapier, 2018). Over time, the maturing heart transitions to metabolizing lipids as a primary source for ATP in a process known as β-oxidation (fatty acid oxidation, FAO). Adult cardiomyocytes preferentially utilize fatty acids, unlike most other cells in the body which typically prefer carbohydrate metabolism (Lionetti et al., 2011). From adolescence to adulthood under normal conditions, the heart utilizes approximately 70–90% fatty acid as its primary metabolic substrate, whereas carbohydrates can provide anywhere between 10–30% of the total acetyl-coenzyme A (CoA) generated (Tuunanen et al., 2008; Martín-Fernández and Gredilla, 2016; Piquereau and Ventura-Clapier, 2018).

Conversely, as the heart approaches old age, instead of utilizing the major metabolite that drives β-oxidation known as fatty acyl-CoA, pyruvate-derived metabolite acetyl-CoA becomes the primary metabolite for energy production (Tang J. X. et al., 2020). Previous reports demonstrated that there is a 40% decline of fatty acid utilization for ATP generation in the aged mammalian cardiomyocyte, however there is no such decline in the proportion of carbohydrates metabolized (Figure 2) (Hansford, 1978; Lakatta and Yin, 1982). One study determined that this switch to glycolysis occurs around the age of 65 in human subjects, a change that may contribute to the lower amounts of ATP associated with longevity (Kates et al., 2003; Dai et al., 2014). The alteration in metabolic behavior of cardiomyocytes from adulthood to old age is understood to be a response to stressful stimuli caused by pathological conditions associated with aging (Picca et al., 2018). Carbohydrate metabolism utilizes a lower amount of oxygen per ATP molecule generated, and therefore may be more advantageous for myocytes during states of hypoxic stress (Ussher et al., 2012). This emphasizes how adaptive mitochondria must be in times of stress in order to maintain ATP production (Stanley et al., 2005). Therefore, the occurrence of a metabolic shift from β-oxidation to glycolysis pathways is a classic cardiac aging phenotype. This causes an imbalance that results in lipid toxicity in the aged cardiomyocyte (Koonen et al., 2007). Indeed, lipotoxicity is a well-established characteristic of an aged myocardium (Slawik and Vidal-Puig, 2006).

Despite the time-dependent decrease in fatty acid utilization, intracellular FA uptake is known to increase upon the rise of sarcolemmal transporter CD36 expression, which is reported to be responsible for 50% of all lipid uptake in the mammalian heart (Koonen et al., 2007; Nagendran et al., 2013). Fatty acid oxidation metabolite acyl-CoA cannot be utilized for energy production until it is internalized into mitochondria by carnitine-palmitoyl transferase-1 (CPT1) (Kolwicz et al., 2013). CPT1 is a rate-limiting enzyme known to be dramatically downregulated in old rat hearts, which may further explain the decrease in oxidative phosphorylation during aging (Long et al., 2012; Zhang X. et al., 2019). Without adequate CPT1 expression to regulate mitochondrial fatty acid uptake, the likelihood of cytoplasmic lipotoxicity increases as these metabolites are unable to be processed, leading to potential contractile dysfunction, cardiac hypertrophy, and eventual heart failure (Sharma et al., 2004; He L. et al., 2012).

In addition, components of the β-oxidation pathway are transcriptionally downregulated in the aged heart due to the decreased expression of their positively regulating transcription factors such as PPAR, retinoid X receptor-α (RXRα), and PPAR gamma co-activator 1 (PGC-1) (Lopaschuk et al., 2010; Dillon et al., 2012). PGC-1 isoforms such as PGC-1α and PGC-1β are originally both highly expressed in the young heart and are considered master regulators of mitochondrial biogenesis; however, cardiac PGC-1α/β expression is commonly reported to decrease during aging (Figure 2) (Dillon et al., 2012). One study observed that the downregulation of PGC-1α and its target gene estrogen-related receptor α (ERRα) are a key features of the failing human heart, an observation which may be involved in the age-related reduction of mitochondrial metabolic capacity (Sihag et al., 2009). Deletion of either isoform in mice contributes to decreased mitochondrial biogenesis, lower mitochondrial volume, and a reduction in both nuclear and mitochondrial encoded genes within heart and skeletal muscle (Arany et al., 2005; Lelliott et al., 2006; Adhihetty et al., 2009). Indeed, genetic ablation of both isoforms (PGC-1αβ^−/−^ mice) leads to extreme phenotypes such as smaller heart size, sudden cardiac arrest, atypical mitochondrial morphology, and a detrimentally low cardiac output, all of which results in low survival beyond a few days post-birth (Lai et al., 2008). These findings confirm that PGC-1 is critical for the energy metabolism and overall health of the adult heart.

Another major attribute of myocardial aging is the harmful decrease in ATP generation with time (Figure 2) (No et al., 2020b). Studies have reported that elderly human and mice mitochondria express reduced levels of electron transport chain (ETC) components (complexes I-V), thus lowering total ATP synthesis rate (Zahn et al., 2006). In states of energy depletion, ketones such as acetoacetate, acetone, and β-hydroxybutyrate can also be utilized as alternate substrates for the generation of energy in cardiomyocytes through a process known as ketone oxidative phosphorylation (ketosis) (Selvaraj et al., 2020). Various reports have established that the metabolic utilization of ketones can therapeutically lessen the maladaptive effects of cardiac aging by prevention of age-associated myocardial remodeling, inhibition of apoptosis in elderly murine myocardium, and contribution of additional oxidative ATP production (Balietti et al., 2009; Sedej, 2018; Yu et al., 2020). Notably, a ketone rich diet was also shown to lengthen the lifespan of adult mice (Roberts et al., 2017). An increase in ketone bodies, specifically β-hydroxybutyrate dehydrogenase 1 (βDH1, also known as βOHB), was observed in murine hearts showcasing classic features of heart failure, suggestive of a shift to metabolizing ketone bodies to generate ATP in these contexts (Aubert et al., 2016). Compared to glucose, βDH1 requires less oxygen for ATP synthesis, which results in less oxidative stress, lower inflammation, and more efficient energy production (Bedi et al., 2016). This may demonstrate that the increase of ketone bodies as a source for energy, much like the metabolic switch from FAO to glycolysis, may be a protective mechanism to reduce the intracellular complications that correlate with age. Nonetheless, there is no current consensus on whether ketosis is truly a cardioprotective mechanism to prolong cardiomyocyte life (Nilsson et al., 2016; McSwiney et al., 2018).

As cardiac metabolism becomes altered over time, responsive enzymes facilitate energy production in order to prevent cardiovascular deterioration. The silent mating type information regulation (SIRT) family of proteins, also known as sirtuins, are key regulators of sustaining metabolic function and protection against myocardial stressors that commonly emerge in the aging heart (Cencioni et al., 2015). As metabolic sensors, sirtuins can sense the energy state of the cell in order to enhance metabolic efficiency as well as mitochondrial function when necessary. Sirtuins can also promote ATP generation, for example SIRT3, which is highly expressed in the heart, increases the enzymatic activity of ATP synthase β, a catalytic subunit of mitochondrial complex V of the ETC, through its deactylation activity (Lombard et al., 2007; Rahman et al., 2014). Indeed, genetic ablation of SIRT3 in the heart leads to decreases in overall ATP synthesis, OXPHOS activity, oxygen consumption, and rates of fatty acid and glucose metabolism (Ahn et al., 2008).

With regards to aging, sirtuins have been observed to ameliorate classic features of myocardial stresses that arise over time. SIRT3 overexpression is protective against age-related cardiac phenotypes (Sundaresan et al., 2009). This protective role of SIRT3 was further confirmed by another study, which determined that SIRT3 activity made aged murine hearts more resistant to I/R-injury (Porter et al., 2014). Genetic deletion of SIRT2 resulted in an accelerated aging phenotype, such as spontaneous cardiac hypertrophy, fibrosis, and overall dysfunction, whereas overexpression of SIRT2 improved viability in cultured myocytes (Sarikhani et al., 2018). SIRT1 expression is considered to be important for maintaining cardiomyocyte health during aging, demonstrated by cardiac-specific SIRT1 overexpression which preserved systolic function and reduced hypertrophy, fibrosis, and senescence marker expression in 18 month old mice (Alcendor et al., 2007).

The ability of sirtuins to act as metabolic sensors is the result of their activation being dependent on nicotinamide adenine dinucleotide (NAD^+^), a redox carrier typically converted to NADH by accepting a hydride group from either glycolysis, TCA cycle, or fatty acid oxidation, which is crucial for driving oxidative phosphorylation (Xie et al., 2020). Unfortunately, there is an age-related decline of NAD^+^ that is well known to contribute to a myriad of aging hallmarks, such as metabolic imbalance, mitochondrial dysfunction, oxidative stress, and pro-inflammatory conditions (Haigis and Sinclair, 2010; Nakagawa and Guarente, 2011). Due to the utilization of NAD^+^ as a substrate for SIRT enzymatic activity, as well as its utilization by other enzymes such as DNA repair enzyme poly (ADP-ribose)-polymerase 1 (PARP-1), the pool of available NAD^+^ decreases within myocardium during aging (Figure 2) (Pillai et al., 2005). Complex I governs the oxidation of NADH to generate NAD^+^, however one study determined that a mouse model of dysfunctional complex I (Ndufs4^−/−^) resulted in a decreased NAD^+^/NADH ratio and heart failure (Karamanlidis et al., 2013), therefore it is hypothesized that complex I dysfunction may occur in aged hearts which prevents the replenishment of NAD^+^ stores (Bugger et al., 2016). Since NAD^+^ is consumed and may not be replenished, it is clear why the sustained imbalance of NAD^+^ levels would exacerbate age-associated depletion of ATP as the metabolic-sensing abilities of sirtuins would be rendered inactive (Xie et al., 2020). Ultimately, the decline in sirtuin activity results in many consequences for the onset and perpetuation of maladaptive cardiac aging.

In summary, there are various forms of mitochondrial bioenergetics that generate ATP in the heart such as the ETC, β-oxidation, glycolysis, and ketosis (López-Otín et al., 2013). As these aforementioned studies have demonstrated, the maintenance of fatty acid oxidation as the predominant source of energy is a characteristic of normal adult heart function and alterations to this metabolic programming can accelerate aging phenotypes. Loss of homeostasis between mitochondrial energy production pathways is due to the altered expression of key regulatory enzymes such as PGC-1 and the sirtuin family, which results in the metabolic remodeling and subsequent energy deficit that occurs in aged hearts. Further investigation is needed to determine the cause of age-related metabolic switching and eventual cardiac decline.

## 4 Increase in mitochondrial ROS generation

Reduction and oxidation (redox), a biochemical reaction that involves the loss or gain of electrons between molecules, is the source of oxidative stress in the heart which leads to age-related pathologies (Cortassa et al., 2021). This transfer of electrons can be mediated by ROS (Fuloria et al., 2021). The mitochondria are the greatest source of ROS within a myocyte, with the majority of ROS resulting from the ETC. Within the ETC, NADH and FADH_2_ are responsible for donating electrons to complex I (NADH dehydrogenase) and complex II (succinate dehydrogenase), which are then transferred to complex III (cytochrome b-c1) by way of ubiquinone [coenzyme Q (CoQ)], then to complex IV (cytochrome c-oxidase), until they reach the final acceptor, molecular O_2_ (Jang and Javadov, 2018). ROS can be produced in various ways, however it is mainly derived from the reverse electron transport through complex I by way of either low CoQ availability or high proton-motive force (Figure 2 **)** (Robb et al., 2018). ROS can also become generated by single electrons escaping the ETC in a passive process known as electron leakage (Zhao et al., 2019). Electrons can interact with and reduce O_2_ to form an extremely unstable superoxide anion (O_2_^−^ or O_2_^•–^) (Kausar et al., 2018). To counteract this form of ROS generation, superoxide dismutase (SOD) initiates the conversion of O_2_^−^ into a more stable form known as hydrogen peroxide (H_2_O_2_) (Li X. et al., 2019). However, H_2_O_2_ is readily interacts with metal atoms to generate a highly reactive hydroxyl radical (OH^•^) (Wang et al., 2020). The common hydroxyl radical (OH^•^) indiscriminately scavenges electrons off biomolecules such as proteins, lipids, and nucleic acids, thus damaging the myocyte.

While it is well established that ROS can be harmful inducers of oxidative stress which contribute to impaired cardiac health, it is important to emphasize that these molecular species are essential for regulating a wide range of physiological phenomena, as long as intracellular ROS concentrations are controlled (Murphy et al., 2011). Mitochondrial-derived oxidants can serve as mediators for a variety of ROS-mediated signaling pathways important for baseline cardiomyocyte function (Shadel and Horvath, 2015). An example of this can be observed from the ability of O_2_^−^ and H_2_O_2_ to promote cardiomyocyte growth and viability (Finkel, 2003; Tsutsui et al., 2009). Furthermore, nitric oxide (NO^•^, NO) has been linked to the regulation of mitochondrial biogenesis, improving OXPHOS function, and ATP content by signaling through its second messenger, 3′,5′-cyclic guanosine monophosphate (cGMP) (Nisoli and Carruba, 2006). NO is synthesized by endothelial nitric oxide synthase (eNOS) and is derived from the endothelium of coronary vasculature as well as from cardiomyocytes (Gödecke et al., 2001). Upon stimulation of eNOS, increases in NO activity enhance ventricular filling capacity by lengthening diastolic intervals and lowering frequency of contractility (Gao, 2010). The contributing factors that determine which oxidation reaction or signaling pathway becomes engaged by mitochondrial ROS depends on the species of ROS generated, radical concentration, duration of oxidant, and the microdomains in which ROS is produced/present (Finkel and Holbrook, 2000).

Oxidative stress can be defined as the functional dysregulation of molecules due to the removal of electrons and introductions of unusual charges into their structures (Sies, 2020). According to the radical theory of aging, which was first proposed in the mid-20th century, a main contributing factor of aging is related to deleterious attacks by free radical species within a cell over time, resulting in additive oxidative stress unto biological structures that drives the functional decline in aged animals (Figure 2) (Harman, 1956). Indeed, previous investigations support this theory as they have demonstrated that there is an increased production of ROS in the aged heart, such as the finding that heart mitochondria isolated from elderly rats (23 months) produced 25% more super-oxide radicals than young rats (3 months) (Nohl and Hegner, 1978). Additional evidence in support of the radical theory of aging is shown by a knock-out mouse model of the p66^Shc^ gene (p66^Shc−/−^), which encodes for a mitochondrial redox enzyme that generates ROS from ETC by-products (Giorgio et al., 2005). The p66^Shc−/−^ mice study determined that targeting this enzymatic source of ROS successfully lengthened the lifespan of these animals by 30% (Migliaccio et al., 1999). Age-related increases of ROS production results in the accumulation of damaged proteins and dysfunctional mitochondria, of which exacerbate stress (Anderson et al., 2018). The increased amounts of oxidized macromolecules and ROS production levels contribute to a multitude of age-associated cardiovascular diseases (Toba and Lindsey, 2019). When this harmful process overwhelms the cardiomyocyte, there are several known protective processes that serve to maintain protein and organelle quality, which will be discussed further in the sections below.

Besides ROS production, oxidative stress also is the result of a decrease in homeostatic antioxidant activity (Balaban et al., 2005; Dai et al., 2012). Biological antioxidants such as glutathione peroxidase or catalase exist in the myocardium to provide endogenous defenses against ROS (Figure 2) (Snezhkina et al., 2019). In one previous study, an overexpression mouse model of human antioxidant catalase (mCAT) increased life span due to lower levels of oxidative stress, and exhibited reduced cardiac aging phenotypes such as enhanced contractile function and mitigated hypertrophy (Schriner et al., 2005). Additionally, mCAT mice exhibited attenuated mitochondrial H_2_O_2_ toxicity, oxidative DNA damage, and mitochondrial DNA (mtDNA) mutation accumulation. Therefore, the findings of this study support the hypothesis that abnormal antioxidant activity is a limiting factor to maintaining health in the aged heart, and highlight a potential in utilizing antioxidants as a therapeutic intervention.

The redox-state of a cardiomyocyte can be determined by taking a ratio of oxidant to antioxidant levels, a balance that relies on the conversion between the oxidized and reduced states (Schafer and Buettner, 2001). Examples of redox couples commonly measured to assess the redox-state of cardiomyocytes are NADH:NAD^+^, cysteine:cystine, and GSH:GSSG (Figure 2). The glutathione redox couple comprises the majority of redox couples in the cell, therefore, this balance of reduced GSH to oxidized GSSG has been utilized as a marker for oxidative stress (Jones, 2002; Wang and Wang, 2021). Once oxidized, GSSG can scavenge electrons from cysteinyl thiols within the structures of various proteins in a reaction known as S-thiolation or glutathiolation (Kouakou et al., 2019). An imbalanced redox state where oxidizing species dominate results in an upsurge of S-thiolation, and consequently, the disturbance of physiological structures and functions of many critical enzymes. A recent study performed an analysis of the effects of hypoxia on redox biomarkers within young and aged Wistar rats and determined that aged hearts were more prone to thiol (T-SH) group oxidation, and thus, more susceptible to oxidative stress (Ağaşcıoğlu et al., 2019). Another study supports the notion that ROS levels are certainly increased in the aged myocardium, as they observed that age-related ROS levels only worsen the damaging effects of I/R injury, resulting in significantly greater mitochondrial dysfunction and cardiomyocyte death (Zhang et al., 2020). Furthermore, sirtuins (SIRT1 and SIRT3) have been associated with efficiently reducing ROS levels in a protective mechanism against I/R stress in aged hearts, since the genetic deletion of SIRT1/3 results in excessive oxidative stress, mitochondrial dysfunction, and cardiomyocyte death (Zhang et al., 2020). Together these studies demonstrate the importance of conserving cardiomyocyte redox state, as disturbed redox homeostasis can lead to various consequences regarding aberrant signaling, mitochondrial dysfunction, and cell death.

The mitochondrion is the only organelle other than the nucleus with its own genetic material. Mitochondrial DNA is circular and comprised of 37 genes, 13 of which encode for essential ETC subunits, housed within the matrix of the mitochondrion (Ryzhkova et al., 2018). Notably, the vast majority of proteins expressed at the level of the mitochondria are nuclearly encoded and are imported to the mitochondria. The occurrence of mtDNA point mutations and deletions have been observed to increase with age in human heart as well as other tissues (Figure 2) (Cortopassi and Arnheim, 1990). An increase in the production of mitochondrial radicals presents a potential risk to mtDNA stability (Lu and Finkel, 2008). One previous report has shown that there is an increase in H_2_O_2_ in SSM and oxidative stress levels in IFM isolated from the hearts of old male rats (24 months) compared to younger rats (6 months) (Judge et al., 2005). As reported in another investigation, the senescence-accelerated mouse model (SAMP8 mice) displayed an age-associated increase in lipid peroxidation products indicative of increase oxidative damage with respect to time (Rodríguez et al., 2007). The phenomenon of age-related increase of ROS-dependent mtDNA damage was highlighted by a previous study in which investigators utilized an oxidative stress biomarker known as 8-oxo-2′-deoxyguanosine (8-OH-dG), a major product of mtDNA oxidation, and measured this biomarker in hearts of eight various mammalian species with a spread of maximal life spans ranging from 3.5 to 46 years (Barja and Herrero, 2000). This study determined that greater amounts of 8-OH-dG were detected within short-lived animals, and vice versa, suggestive that excessive mtDNA damage correlates with shortened lifespans (Barja and Herrero, 2000). Overall, it is apparent that oxidative damages upon mtDNA have been shown by various sources to correlate with increasing age. Further research has shown that a mouse model of a proof-reading deficient version of mitochondrial polymerase PolgA (mtDNA mutator mouse, PolgA^mut/mut^) caused various premature aging phenotypes such as reduced lifespan, weight loss, alopecia, and heart enlargement (Trifunovic et al., 2004). Despite this, recent reports have suggested that mtDNA deletions, not mtDNA point mutations, serve as the driving force behind the shortened lifespan observed in these mice (Vermulst et al., 2008). In contrast to this argument, other researchers hypothesize that mtDNA replication errors made by mtDNA polymerases are the driving factor in aging instead of ROS-dependent mtDNA mutations (Kauppila et al., 2017). It is also important to note that the mutator mouse model has been challenged since these animals experience greater levels of mtDNA mutations than aged humans, and therefore do not recapitulate the natural aging processes (Khrapko et al., 2006). Additionally, previous research efforts have shown that there are very low levels of specific mtDNA deletions attributed to the phenomenon of aging (Cortopassi and Arnheim, 1990). It is currently debated whether mtDNA mutations directly cause aging or simply correlate with the process (Vermulst et al., 2007). Therefore, the origin of mtDNA mutations and their contributions to the cardiac aging process remains to be fully elucidated.

At baseline, mtDNA has a higher mutation rate than nuclear DNA due to the high presence of radical species in the local microenvironment (Hu et al., 2020). One mathematical model demonstrated that 90% of cardiomyocytes will undergo a minimum of 100 mtDNA mutations by the age of 70, which likely contributes to age-related mitochondrial dysfunction (Li H. et al., 2019). Although the nuclear genome is not impacted as severely, poor nuclear genome stability can occur in the instance of mitochondrial dysfunction, since imbalanced ROS-to-antioxidant mode of oxidative stress can eventually surpass the local microenvironment and induce an increase in nuclear mutations as well (Veatch et al., 2009). In addition to SIRTs metabolism-related functions, as previously mentioned in this review, this family of deacetylases can also increase cardiomyocyte levels of mitochondrial antioxidants such as superoxide dismutase 2 (SOD2) in the event of detrimental oxidative stress (Figure 2) (Imai and Guarente, 2014). Sirtuins can also engage in nuclear as well as mtDNA damage repair as they can induce deacetylation reactions directly upon hyper-acetylated chromatin or correct DNA repair proteins (Tennen and Chua, 2011).

In summary, there is a critical need to address the excessive generation of radical species during aging. Due to the nonselective damage that occurs due to oxidative stress, radical species and antioxidants both serve as a major therapeutic targets to deaccelerate age-associated cardiac phenotypes. Additional research must be implemented to reduce the oxidative burden of the old heart, and therefore preserve mtDNA stability, sustained mitochondrial membrane integrity, and maintain a balance of the overall redox state of the cardiomyocyte. However, therapeutic attempts to reduce the burden of ROS to prevent age-related decline have had little success as discussed in section 7.

## 5 Decline in mitophagy during aging

Since mitochondria are the central generators of ROS within the cardiomyocyte, they are constantly faced with the threat of oxidative damage that can lead to the functional failure of the organelle (Onishi et al., 2021). In this event, there is a need for these dysfunctional organelles to be cleared from the cell to preserve viability (Scheibye-Knudsen et al., 2015). Dysfunctional mitochondria can be selected for degradation in a sub-type of autophagy that is known as mitophagy, termed for its mitochondrion-specific engulfment (Kubli and Gustafsson, 2012). Currently, there are two main forms of this mitochondrial quality control mechanism that exist in the mammalian heart: 1) ubiquitin-dependent mitophagy, and 2) receptor-mediated mitophagy (Ding and Yin, 2012; Jin and Youle, 2012).

Ubiquitin-dependent mitophagy is regulated by phosphatase and tensin homolog (PTEN)-induced putative kinase protein 1 (PINK1) and Parkinson Protein 2 (Parkin). PINK1 is targeted to the mitochondria, while Parkin is a cytosolic protein. Under normal conditions, PINK1 activity remains negatively constrained by mitochondrial processing peptidase (MPP) and presenilin-associated rhomboid-like protease (PARL) via consistent degradation through cleavage (Jin et al., 2010). Upon the loss of mitochondrial membrane potential, which is a classic feature of mitochondrial dysfunction, PINK1 will translocate on the OMM and mediate the subsequent translocation of Parkin, an E3 ubiquitin ligase, to the dysregulated mitochondrion (Matsuda et al., 2010). Parkin will then ubiquitinate outer membrane substrates such as voltage-dependent anion channel (VDAC), translocase of the outer membrane (TOM), mitofusins, BAK, and more (Sarraf et al., 2013). Autophagy adaptors such as optineurin (OPTN) or p62/SQSTM1 utilize their ubiquitin-binding domains (UBD) to identify these ubiquitinated substrates, and consequently recruit microtubule-associated protein light chain 3 (LC3) to enable autophagosome engulfment of the mitochondrion for fated lysosomal fusion and degradation (Ajoolabady et al., 2020).

Receptor-mediated mitophagy is mediated through mitophagic receptors on the OMM such as FUN14 domain containing 1 (FUNDC1), Bcl-2-like protein 13 (Bcl2L13/Bcl-Rambo), FK 506-binding protein (FKBP8), Bcl2/adenovirus E1B 19-kDa interacting protein 3 (BNIP3), and NIX/BNIP3L (Novak et al., 2010; Hanna et al., 2012; Liu et al., 2012; Murakawa et al., 2015; Bhujabal et al., 2017; Wei et al., 2017). All these receptors are anchored to the OMM and bind directly to LC3 to guide its tethered organelle to the autophagosome, bypassing the need for ubiquitination (Liang and Gustafsson, 2020). Another receptor, Prohibitin-2 (PHB2), found within the IMM also binds with LC3, but this event typically occurs when the outer membrane is permeabilized or ruptured (Wei et al., 2017). Each of these mitophagy receptors can bind to LC3 for the sequestration of mitochondria by their LC3-Interacting-Regions (LIRs), which are conserved domains located at various sites specific to each regulator. Although these receptors are established, their mechanisms of activation are still not well understood (Liang and Gustafsson, 2020). Thus far, it is understood that some of these receptors are regulated by phosphorylation status to become activated and bind LC3.

It is interesting to note that BNIP3 and Bcl-Rambo are both members of the Bcl-2 family of apoptotic regulators, based on the presence of Bcl-2 homology (BH) domains within their peptide structures. However unlike BNIP3, the role of Bcl-Rambo in apoptotic cell death remains to be controversial (Meng et al., 2021). Containing all four BH domains (BH1-4) Bcl-Rambo has been shown to either induce cell death upon overexpression into HEK 293T cells, or promote cell viability as in glioblastoma cancers, resulting in controversial conclusions within the field (Kataoka et al., 2001; Jensen et al., 2014). Despite this ambiguity in apoptosis, newly emerging studies have demonstrated that Bcl-Rambo promotes mitochondrial fission as a precursor to mitophagy (Murakawa et al., 2015). It has yet to be determined whether Bcl-Rambo is protective or maladaptive with respect to the aging heart. BNIP3 is a BH3-only protein that is typically inactive during physiological conditions, yet upon stressful stimuli such as hypoxia, it has been previously associated with the induction of cell death, since its activation has been shown to correlate with a loss of membrane potential, and an increase in oxidative stress (Burton and Gibson, 2009). Once activated BNIP3 becomes upregulated and translocates to the OMM as a stabilized homodimer, anchored by its C-terminus domain (Hanna et al., 2012). Interestingly, a recent study demonstrated that NIX (BNIP3L) and FUNDC1 are critical for stimulating mitophagy in adult cardiac progenitor cells (Lampert et al., 2019). Overall, the mitophagic functions of these Bcl-2 proteins demonstrates that there are various processes involved with this class of proteins beyond their influences on cell death.

There is an accumulation of dysfunctional mitochondria within the aged myocyte which may be due to the loss of efficient mitophagy in the old heart (Figure 2) (Linton et al., 2015). Not only are defective mitochondria of the aged myocyte not sufficiently degraded, a majority of mitochondria become structurally enlarged and exhibit reduced dynamics such as fission, resulting in an increasing number of these organelles that are poorly engulfed by the autophagosome (Terman et al., 2010). One study demonstrated that there is a decrease in PINK1 protein expression in the hearts of middle-aged to elderly human patients with end-stage heart failure when compared to PINK1 levels in healthy controls (Billia et al., 2011). Mitophagy was also determined to be diminished in old hearts according to another study, where investigators subjected old vs. young WT mice to a mitochondrial uncoupler to induce a mitophagic response (Hoshino et al., 2013). As a result of this study, investigators observed that mitochondria within the hearts of adult WT mice (10 months) appeared to be encapsulated within autophagosome vacuoles more so than the mitochondria of aged mice (20 months) (Hoshino et al., 2013). Evolutionarily conserved cysteine residues on mitophagy regulator Parkin are prone to oxidation by ROS, resulting in the loss of Parkin’s ubiquitin ligase activity, misfolding, and subsequent clearance from the cell (Wong et al., 2007; Meng et al., 2011). Therefore, it has been postulated that the excess of oxidative stress, a classic feature of the aged myocardium, may decrease the efficiency of Parkin-mediated mitophagy within the heart (Liang and Gustafsson, 2020). Unfortunately, less is known about the status of the mitophagy receptors during aging, although BNIP3 is known to be upregulated in aged hearts (Lee et al., 2011). Interestingly, BNIP3 and Nix are thought to become maladaptive cell death inducers during age-related myocardial stresses such as heart failure and I/R injury, however this harmful shift from promoting mitochondrial quality control is yet to be fully understood (Hamacher-Brady et al., 2007; Baines, 2010; Shires and Gustafsson, 2015).

Pharmacological or genetic alterations of the mitophagy pathway influences aging. Chemically inducing mitophagy by natural compound, urolithin A (UA), can lead to the clearance of damaged mitochondria and extend the lifespan of *C. elegans* (Ryu et al., 2016). Importantly, this result has been translated into the mouse model system through orally consumed UA which protects against age-related muscle decline (Ryu et al., 2016). Additionally, Parkin-deficient mice (Parkin^−/−^) exhibit features of accelerated cardiac aging such as accumulations of dysfunctional mitochondria within their hearts and these mice experience exaggerated cardiac damage and increased mortality when stressed (Kubli et al., 2013). Conversely, Parkin overexpression in mice delays cardiac aging and improves mitochondrial health (Rana et al., 2013). Moreover, cardiac-specific Parkin overexpression leads to a decrease in abnormal mitochondria, as well as a resistance to an age-dependent decrease in oxygen consumption (Hoshino et al., 2013). However, Parkin overexpression leads to an increase in cardiac fibrosis (Woodall et al., 2019), perhaps due to the over activation of the autophagic pathway. This suggests, while increasing the rate of mitophagy is protective, there is a threshold where too much may be maladaptive. Interestingly, transgenic mice that accumulate mtDNA mutations caused by defective mtDNA polymerase were not protected by cardiac-specific overexpression of Parkin (Woodall et al., 2019). This may demonstrate that there is a minor role of Parkin-dependent mitophagy in cardiomyocytes, at least concerning mitochondrial instability genomic driven dysfunction. Unfortunately, less is known about the status of the mitophagy receptors during aging, although BNIP3 is known to be upregulated in aged hearts (Lee et al., 2011).

A powerful genetic tool used as a reporter of mitophagy is the mtKeima transgenic mice model, which express a pH-sensitive and lysosome resistant-protein known as Keima which possesses dual-excitation fluorescence (Sun et al., 2017). Fluorescent Keima protein originates from coral and is localized to mitochondria using the mitochondrial targeting sequence of inner-membrane subunit Cox VIII (Sun et al., 2015). When at neutral pH within uncompromised mitochondrial, Keima becomes excited at approximately 458 nm recognized as green fluorescence, yet upon co-localization with the lysosome which contains an acidic pH of 4.5, the probe gradually shifts its excitation wavelength to 561 nm which appears as red (Sun et al., 2017). Using this novel genetic tool, researchers have shown that both the heart and brain contain higher levels of baseline mitophagy compared to the other tissues in the body (Sun et al., 2017). In the future, these mice will be a valuable asset to investigate changes in mitophagy that occur during aging. Another group engineered a mouse model known as the αMHC-MitoTimer mice, which allows investigators to identify mitochondrial turnover with respect to the passage of time (Stotland and Gottlieb, 2016). This was accomplished by using MitoTimer, a mitochondrial-targeted protein which can stably induce cardiomyocyte mitochondria to fluoresce from green-to-red over a course of several hours, all of which was driven by a cardiac driven alpha-MHC promoter (Williams et al., 2017). After comparing the hearts of young (3 weeks) and old (16 weeks) αMHC-MitoTimer mice, investigators observed a higher population of green-fluorescent mitochondria in young hearts compared to older littermates, which demonstrated that the rate of mitochondrial degradation was decreased in the aged cohort (Stotland and Gottlieb, 2016). In all, the decline in efficient mitochondrial clearance from the myocyte is one of many central features of cardiac aging and further studies must be conducted to re-establish quality control of this vital organelle.

## 6 The decline of autophagy and the ubiquitin-proteasome system

It is critical for the non-proliferative cardiomyocyte to maintain adequate protein quality control, which entails the regulation of proper protein translation, folding, trafficking, localization, and degradation. The quality control pathways which occur commonly within the myocardium are autophagy and the ubiquitin-proteasome system (UPS), which are lysosomal- and proteasomal-dependent, respectively (Sun-Wang et al., 2020). Here in this section we will review previously published research of these protein degradation mechanisms with special emphasis on the implications of the aged myocardium.

Autophagy is a mechanism important for maintaining the quality of proteins and organelle systems by selective lysosome-dependent degradation of defective intracellular components (Saha et al., 2018). There are three types of autophagy known as macro-autophagy, micro-autophagy, and chaperone-mediated autophagy. However, here we will focus primarily on macro-autophagy as it is the most widely characterized form that exists within the heart (Yamaguchi, 2019). Autophagy can serve either protective or maladaptive roles when upregulated in response to a stressful event, dependent on the pathological context (Sciarretta et al., 2012). The functional unit of autophagy is the autophagosome, a double lipid membrane that encapsulates cargo destined for degradation once it fuses to a lysosome. The initiation of autophagy begins with the seeding of an isolated membrane known as the phagophore. Phagophore formation is controlled by two multi-protein complexes, the Atg13/ULK-1/Atg101/FIP200 heterotetratmer which is regulated tightly by metabolic sensors mammalian target of rapamycin complex 1 (mTORC1) and AMP-activated protein kinase (AMPK) (Alers et al., 2012). Once the ULK1 complex forms at designated autophagophore assembly sites, it initiates phagophore formation by the recruitment and subsequent activation of the class III phosphoinositide 3-kinase (PI3K) complex I which is composed of Vps34, Vps15, Beclin-1, and Atg14 (Russell et al., 2013). The PI3K complex is responsible for producing phosphatidylinositol 3-phosphates (PI3Ps) which recruits yet another complex known as the ATG2-WIPI complex (Al-Bari and Xu, 2020). The phagophore can expand in size as further regulatory proteins are recruited at the assembly site such as the ubiquitin-like proteins that behave like E1 (ATG7), E2 (ATG10 and ATG3), and E3 (ATG12-ATG5-ATG16 L complex) UPS enzymes (Geng and Klionsky, 2008). Altogether, these complexes complete the maturation of the phagophore into the nascent autophagosome by way of ATG4-induced conjugation of LC3-I (ATG8) to phosphatidylethanolamine (PE) to form LC3-II (ATG8-II) (Geng and Klionsky, 2008). The lipidation of cleaved LC3-I to form LC3-II is critical for autophagy, hence, LC3-II is a common marker of autophagy (Ghosh and Pattison, 2018). Adaptor proteins such as p62/SQSTM1 link the damaged cargo to LC3-II using ubiquitin-associated domains and LIRs, respectively (Gatica et al., 2018; Johansen and Lamark, 2020). This process finalizes once the autophagic machinery fully engulfs damaged cargo and undergoes fusion with the lysosome, causing all sequestered contents to become degraded by lysosomal enzymes (Ravikumar et al., 2010). Amino acids can then be released into the cytosol for nutrient recycling through the amino acid transporter SLC38A9 and Rag GTPase-Ragulator complex which each exist on the lysosomal membrane (Wyant et al., 2017). The release of essential amino acids from the lysosome ensures the continuous growth and survival of the cell.

The UPS mediates the non-lysosomal degradation of damaged or mutated proteins (Zheng and Shabek, 2017). This mechanism initiates once ubiquitin-activating enzyme (E1) activates ubiquitin in an ATP-dependent manner, then ubiquitin conjugation enzymes (E2) attach multiple ubiquitin molecules to the lysine residues of abnormal proteins fated for degradation (Akutsu et al., 2016). After further interaction by E3 ligase enzymes, which acts as a scaffold for the targeted substrate and the E2-ubiquitin complex to achieve a significant ubiquitination status (Pohl and Dikic, 2019). The ubiquitinated protein is then ready for digestion by the 26S proteasome (Roos-Mattjus and Sistonen, 2004). The 26S proteasome is a 2.5 MDa multi-protein complex that consists of a 33 subunit assembly known as the 20S core particle which is capped by the 19S regulatory particle (Rousseau and Bertolotti, 2018). During physiological conditions, UPS is an ATP-dependent quality control mechanism, yet in times of pathological cardiac stress, the 26S proteasome can perform degradative actions in an ATP-independent manner (Kumar Deshmukh et al., 2019). The UPS system responsible for the majority of intracellular protein maintenance via degradation, as approximately 80–90% of all dislocated, misfolded, oxidized, and dysfunctional proteins become eliminated through UPS-mediated clearance (Bhattacharyya et al., 2014). Therefore, UPS is a highly efficient mechanism that maintains cardiomyocyte health. It is important to note that while the UPS mechanism shares similarities with the autophagic pathway such as a similar goal and the use of ubiquitin-type enzymes, the proteasome targets more transiently-active proteins such as tumor suppressor p53, whereas the lysosome degrades larger, more long-lived proteins such as the myosin heavy chains and entire organelles that cannot fit within the confines of the proteasome core (Zhang et al., 2016).

The UPS system has been established to participate in the turnover of proteins which exist on the OMM (Xu et al., 2011). Originally it was proposed that proteins contained within the mitochondria were not degraded by the UPS, but instead by mitochondrial proteases, due the inaccessibility of the UPS system (Langer and Neupert, 1996). However, this hypothesis has been recently challenged by emerging evidence suggesting that intramitochondrial proteins undergo ubiquitination (Udeshi et al., 2013). It is important to note that 62% of the mitochondrial proteome is ubiquitinated in humans, with a majority of these proteins localized within the matrix and IMM (Lehmann et al., 2016a). One group of investigators determined that degradation of internally housed mitochondrial proteins can indeed be UPS-dependent, since they observed that pharmacological inhibition of the cytosolic 26S protesome led to the increase in levels of a IMM protein known as uncoupling protein 2 (UCP2) (Azzu and Brand, 2010). Other known matrix and IMM proteins have also been hypothesized to undergo UPS-associated proteolysis (Lehmann et al., 2016b; Lavie et al., 2018). The mechanism by which inner membrane and matrix proteins can be degraded has yet to be fully elucidated, however one potential hypothesis is the mitochondria-associated degradation (MAD) pathway, which lead to the extraction of these proteins out of the organelle by Cdc48 (p97/VCP in higher eukaryotes) for degradation by the proteasome (Franz et al., 2014; Liao et al., 2020). Other than the shuttling of intramitochondrial proteins to the proteasome, the proteasome can also become recruited to the mitochondrial outer membrane via anchoring onto FK506-binding protein 38 (FKBP38) (Nakagawa et al., 2007). Further studies must be conducted to fully comprehend the extend of UPS-dependent degradation of mitochondrial contents with respect to aging.

According to a recent report, there are 87 human UPS components including the ubiquitination machinery that reside at the level of the mitochondria, many of which contain mitochondrial targeting sequences and undergo interactions with a variety of non-UPS mitochondrial proteins (Lehmann et al., 2016a). Notably, UPS components localized to the mitochondria are E3 ubiquitin ligase, membrane-associated RING-CH E3 ubiquitin ligase 5 (MARCH5), and deubiquinases ubiquitin specific protease 30 (USP30) and Ataxin-3 (Lehmann et al., 2016a). Notably, the UPS is known to be heavily involved in mitochondrial protein degradation (Neutzner et al., 2008). The UPS can regulate mitochondrial morphological processes such as fission and fusion, as depletion of USP30 expression results in a hyper fusion mitochondrial morphology (Nakamura and Hirose, 2008). Additionally, the UPS system can alter fission and fusion proteins directly by leading to the degradation of Mitofusin 1 (MFN1) or dynamin-regulated protein-1 (DRP1) (Wang et al., 2011). Interestingly, Parkin induces UPS activity to degrade fusion machinery in order to induce mitochondrial fission for the promotion of mitophagy (Tanaka et al., 2010; Chan N. C. et al., 2011). Therefore, the UPS is a highly efficient mechanism that maintains cardiomyocyte health through direct protein clearance and mitochondrial quality control regulation (Heo and Rutter, 2011; Ikeda et al., 2014).

One main feature of cardiac aging is the accumulation of misfolded proteins and dysfunctional organelles due to failed quality mechanisms, a pathological disorder known as cardiac proteinopathy (Su and Wang, 2009). Quality control pathways in the aged heart become severely hampered, resulting in protein toxicity within cardiomyocytes (Tannous et al., 2008). There is an increasing body of evidence suggesting that proteostasis becomes detrimentally altered with age, due to nonfunctional autophagy and/or UPS mechanisms which cannot accommodate the rate of protein synthesis (Löw, 2011). One such study supports the existence of age-related cardiac proteinopathy by analyzing the hearts of old mice where they observed an accumulation of many proteins compared to younger murine cohorts, such as more sarcomeric protein (α-actin), myosin heavy chain beta (β-MHC), B-type natriuretic peptide (BNP), and Atrial Natriuretic Peptide (ANP) (Castello et al., 2010). With an increase in protein content there is greater opportunity for oxidants to target the crowded intracellular landscape, driving further dysregulation (Petropoulos et al., 2000). There is evidence of human cardiac proteinopathy, as amyloid aggregates of small heat shock protein α-B-crystallin and desmin are present in middle-aged human heart samples diagnosed with dilated cardiomyopathy and hypertrophic cardiomyopathy (Sanbe et al., 2004). In summary, it is well established that aging and age-associated diseases such as CVD are strongly correlated with cardiac protein toxicity.

As hearts age, autophagic flux decreases, which further exacerbates cardiomyocyte drop-out and organ failure (Shirakabe et al., 2016). Growing evidence showcases the anti-aging properties of autophagy, as several studies observed that restoring autophagic flux in mouse models of aging or maladaptive protein folding positively improves heart function (Zheng et al., 2010; Bhuiyan et al., 2013; Dutta et al., 2014). Transgenic mice and isolated fibroblasts that overexpress Atg8 experience improved motor function, are more tolerant to oxidant stress, less susceptible to cell death, and have extended lifespans (Pyo et al., 2013). Additionally, the hearts of these Atg8 overexpression mice yielded greater levels of autophagic activity as the mice matured with age compared to WT mice. This would indicate that heightened autophagy levels in the aged heart is indeed cardioprotective. If the process of autophagy is blocked in the myocardium, as observed in an investigation which utilized a Atg5 null mouse, aging phenotypes such as the accumulation of dysfunctional cellular components become accelerated (Nakai et al., 2007). Furthermore, hearts that are Atg5-deficient showcase disorganized sarcomere structure and mitochondrial disorganization. Furthermore, upon pressure-overload stress, the hearts had an increase in dilated hypertrophy of the left ventricle (Nakai et al., 2007). Altogether, these findings suggest that autophagy plays a protective role in the heart, both at baseline and during stress conditions that emerge with aging. Development of autophagy-enhancing interventions may be beneficial for prolonging the function of the heart into old age.

There is also supporting evidence for the necessity of the UPS in the aging heart. Interestingly, proteasome inhibitor drug treatments administered to cancer patients cause adverse cardiotoxicity, which indirectly suggests the importance of the UPS system in the heart (Drews and Taegtmeyer, 2014). In fact, proteasome activity was previously observed to become downregulated in the epidermal cells of elderly human subjects (Petropoulos et al., 2000), as well as in the hearts of rodents (Bulteau et al., 2002). Within the latter study, investigators observed reduced levels of proteasomal 20S core particle in old Fisher 344 rat hearts, which was hypothesized to contribute to enhanced susceptibility of the aging heart to cardiovascular disease (Bulteau et al., 2002). A recent investigation demonstrated that myocardial 26S proteasome activity and UPS proteolytic function could be restored in cardiac proteinopathy-prone mice (CryAB^R120G^) by inhibition of the cyclic nucleotide phosphodiesterase 1 (PDE1), suggestive that the PDE1 enzyme may play a prominent role in proteasome degradation in the aged murine heart (Zhang H. et al., 2019). As for the aged human heart, one study isolated peripheral blood lymphocytes samples from elderly donors and determined that there is an increase in the number of post-translational modifications of individual proteasome subunits, which contributed to age-related decline of 26S proteasome-specific activity (Carrard et al., 2003).

Overall, these findings strongly suggest that aberrant alterations to the function, structure, and activity of the autophagic and proteasomal processes leads to the aging process. In summary, autophagy and UPS pathways are critical for preserving the integrity of the cardiomyocyte. Further understanding of how these pathways are commonly dysregulated during the aging process is needed in order to determine how to best therapeutically intervene.

## 7 Reduction in mitochondrial biogenesis

Due to evidence that mitochondria are susceptible to accruing damage during aging, it becomes critical to maintain mitochondrial function by renewing the mitochondrial pool during aging (Van Remmen and Richardson, 2001). Therefore, mitochondrial turnover must be monitored closely by the cell to ensure healthy progression of energy production, regulation of mitochondrial cell death factors, control of radical oxidative species, and proper housekeeping of the mitochondrial genome (López-Lluch et al., 2008). The generation of new mitochondria is referred to as mitochondrial biogenesis. The dynamic event of biogenesis is closely linked to the processes of growth and division, or fission and fusion, of mitochondrial bodies (Liu et al., 2009). Fusion is accomplished by OMM regulators such as MFN1 and MFN2 and the IMM regulator Optic atrophy 1 (OPA1), which have been shown to be necessary for mitochondrial health (Chen et al., 2011; Piquereau et al., 2012). The genetic deletions of cardiac specific MFN1 and MFN2 leads to a rapid progression of cardiomyopathy (Chen et al., 2011). A major regulator of fission is GTPase DRP1, and its genetic deletion causes an accelerated aging phenotype (Ikeda et al., 2015). Fission typically precedes the breakdown of organellular components (i.e. mitophagy) which occurs in parallel with mitochondrial content synthesis (i.e. protein translation). Therefore, both mitochondrial fission and fusion are required to maintain a healthy population of mitochondria within the heart and the inhibition of either pathway has the potential to accelerate aging. Together, synthesis and degradation events determine the total mitochondrial number within the cell, however, total mitochondrial quantity is not necessarily indicative of a change in mitochondrial biogenesis. Actually, it is solely an increase in the rate of mitochondrial synthesis that is the most reflective measure of mitochondrial biogenesis (Miller et al., 2012). The best method for measuring mitochondrial renewal is to determine the rate of mitochondrial protein synthesis (mitoPS), which is accomplished by the measurements of mitochondrial phospholipid levels or incorporating stable isotopic tracers such as stable isotopically labeled water (^2^H_2_O) into mitochondrial protein precursors (Robinson et al., 2010). While some investigators have utilized mtDNA and mitochondrial mRNA contents as indicators of biogenesis, these methods are not considered to be as accurate as mitoPS, since transcript number does not necessarily equate to total mitochondrial number (Miller et al., 2012).

Unfortunately, it has been established that the rate of mitochondrial biogenesis is greatly reduced in the elderly population (Figure 2) (López-Lluch et al., 2008). One study claimed that the diffusion of H_2_O_2_ and NO^•^ contribute to decreased mitochondrial biogenesis in aged rodents (Navarro and Boveris, 2007). In order to provide the protein and lipid substrate necessary for the generation of new mitochondria, reliable crosstalk between the mitochondria and nucleus must occur, which is mediated by various metabolic sensors and transcriptional regulators (Nirwane and Majumdar, 2018). PGC-1α is known as the master regulator of mitochondrial biogenesis (Steinberg and Kemp, 2009). PGC-1α, which is highly expressed in the heart, serves as a sensor for metabolic changes and/or stressors in order to modify gene expression, and thus reflects the energetic demands of the cardiomyocyte (Fernandez-Marcos and Auwerx, 2011). The regulators of PGC-1α activity are AMPK and SIRT1, both of which are considered to be critical metabolic sensors (Nirwane and Majumdar, 2018). It is well established in the literature how AMPK and SIRT1 directly target transcription factor fork head box O3 (FoxO3) to increase PGC-1α expression (Brunet et al., 2004; Greer et al., 2007). Upon successful activation of PGC-1α via SIRT1-dependent deacetylation, it shuttles from the cytoplasm to the nucleus where it co-activates transcription factors such as PPARα to promote gene expression of various mitochondrial enzymes critical for mitochondrial β-oxidation medium-chain acyl-CoA dehydrogenase (MCAD) (Fernandez-Marcos and Auwerx, 2011). Interestingly, SIRT1 and PGC-1α participate in nuclear-mitochondrial crosstalk for the stimulation of mitochondrial turnover, supported by their dynamic subcellular co-localization and shuttling between the two organelles (Aquilano et al., 2010; Aquilano et al., 2013). While it is important to acknowledge the key role that PGC-1α plays with regard to mitochondrial biogenesis, it is not sufficient to initiate this process alone (Miller and Hamilton, 2012). In summary, stimulating mitochondrial biogenesis could have great effects on maintaining cardiomyocyte longevity. Indeed, several interventions such as caloric restriction and exercise, discussed in the following section, have been shown to induce this process and effectively increase lifespan.

## 8 Preservation of mitochondrial function and heart health

A multitude of efforts have been employed to lessen the severity of age-associated myocardial dysfunction (Giannuzzi et al., 2003; Haykowsky et al., 2007). It is important to note that exercise can have either positive or negative effects on the heart dependent on the intensity, training type, or health of the individual (Haykowsky et al., 2007; Fagard, 2011). Some individuals may have greater risk in developing hypertension, high blood pressure, and other cardiomyopathies based on genetic factors and lifestyle. When utilized efficiently and appropriately to the individual, exercise can become a tremendous asset to conserving heart health (Fagard, 2011).

**FIGURE 3:**
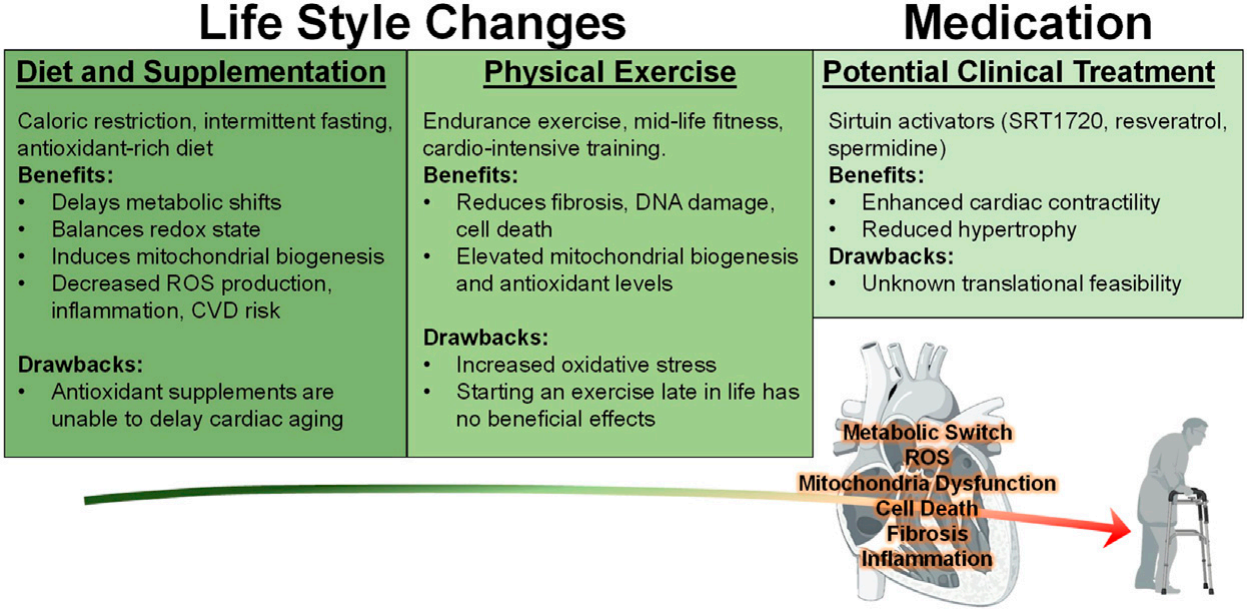
Behavioral and Medicinal Interventions against Cardiac Aging. Currently, various studies have implemented many approaches to reduce the negative effects associated with the aging heart. Diets such as caloric restriction (CR), intermittent fasting (IF), and increasing antioxidant rich foods or supplements have been investigated with regard to heart health. However, the latter was shown to be ineffective at mitigating the effects of aging. On the other hand, CR and IF are both effective at reducing many of the negative consequences of aging. Exercise has been demonstrated to enhance cardiac function in both mouse and human models, however it only produces beneficial effects when it is initiated during middle age and may impose an increased oxidative stress burden. In addition to these lifestyle changes, medicinal intervention utilizing sirtuin stimulators SRT1720, resveratrol, and polyamine spermidine have exerted positive effects against cardiac stress.

Surmounting amounts of evidence show that exercise can indeed improve cardiac aging phenotypes such as cardiomyocyte senescence, telomere shortening, and cell viability (Figure 3) (Werner et al., 2008). In fact, experts recommend that individuals begin engaging in exercise at least at middle-age, since a lack of mid-life fitness is strongly correlated with increased late-life morbidity, hospitalization rate, and a greater risk for heart failure (Pandey et al., 2015). However, initiating an exercise regimen late in life is no longer beneficial, as one investigation demonstrated that 1 year of vigorous trainingstarting at ∼70 years old does not improve negative cardiac remodeling compared to sedentary seniors (Fujimoto et al., 2010). All subjects in this study underwent Doppler echocardiography and maximal exercise testing which led researchers to determine that the long-term period of exercise imparts only minor LV remodeling and favorable, yet insignificant, effects on arterial function and oxygen capacity. Altogether these data solidify the notion that the beneficial effects of exercise can be achieved if initiated by mid-life but not late in life.

Previous findings have shown that exercise-training can elicit an elevated autophagic response in skeletal muscle and cardiac tissue (He C. et al., 2012; Jamart et al., 2012). This is speculated to occur in a BCL-2-dependent mechanism, as it was determined that mutating three critical phosphorylation sites (Thr 69, Ser 70 and Ser 84) within the non-structured loop of murine BCL-2 led to a lack of stimulus-induced, but not baseline autophagy (He C. et al., 2012). Similarly, another study observed that mice subjected to wheel-running over a period of 7 months increased autophagic flux in the heart and improved left ventricular function (Bhuiyan et al., 2013). These seminal findings continue to be upheld by current data: one recent study confirmed how cardiac autophagy and mitophagy can be re-established by exercise-training in a post-myocardial infarction-induced heart failure animal model (Campos et al., 2017), whereas another group determined that a swimming regime can reduce myocardial fibrosis and apoptosis in aged mice hearts (Zhao et al., 2018). Swimming was also able to reduce isoproterenol-induced cardiac injury in mice compared to sedentary mice (Jimenez et al., 2011). One group utilized the mtDNA mutator mouse, which exhibits a lack of proofreading mtDNA polymerase, resulting in a premature aging phenotype (Safdar et al., 2016). However, these investigators observed that this aging phenotype was reversed upon subjecting the mice to endurance exercise. Not only were oxidative stress levels reduced in these exercised-mice, but levels of mitochondrial biogenesis increased. These findings imply that exercise can prevent nuclear DNA damage through a reduction in ROS-induced stress.

Indeed, endurance performance is greatly dependent on mitochondrial fitness and biogenesis, since their respiratory function lies at the root of the maximal oxygen consumption capacity of an individual (Bishop et al., 2019). A recent study demonstrated that PGC-1, a transcriptional regulator of genes important for energy production and mitochondrial biogenesis, is upregulated in rat hearts upon exercise training (Kianmehr et al., 2020). Additionally, mitochondrial biogenesis can be effectively stimulated by various exercise regimens, such as single-leg cycling which was shown to increase mitochondrial content in skeletal muscle compared to the untrained leg within the same subject model (MacInnis et al., 2017). However, there is no general consensus on whether high intensity exercise can reliably induce mitochondrial biogenesis in old age (Bishop et al., 2019).

Twelve week old mice (middle age) subjected to chronic exercise training, resulted in elevated antioxidant glutathione activity in the myocardium (Kakarla et al., 2005). However, exercise only worsened the outcome of oxidative stress, as levels of lipid peroxidation were also observed to increase (Figure 3). This study demonstrated that chronic physical exercise is not sufficient to protect the aged heart from oxidative stress. It is likely that the increase in oxygen consumption and rise in metabolic rate that occurs during exercise could promote ROS activity at the level of lipid membranes, causing their dysregulation. Therefore, doubt has been cast over the hypothesis that exercise-induced antioxidant stimulation can help aged hearts cope with the harmful load of free radicals. The pros and cons of exercise must be considered to better understand the extent of the benefits it can provide for the aged heart.

Similar to exercise training, specific forms of dietary training have been proposed to contribute to increased health benefits. Caloric restriction (CR), which is the limited dietary intake of calories, has demonstrated positive outcomes against aging. Multiple studies demonstrate a strong link between over-eating and cardiovascular disease, since strict dietary restrictions in human patients successfully lowers blood pressure, serum lipids, and maintains vascular health (Holloszy and Fontana, 2007). Contrary to popular belief, the combination of CR and exercise was shown to not provide an additive benefit in lowering total body fat or enhancing cardiovascular health (Redman et al., 2007). Instead, the induction of an exercise regimen is equally as beneficial compared to restricting caloric intake with nearly identical outcomes, despite the enhancement of aerobic fitness provided exclusively by exercise (Redman et al., 2007).

According to recent reports, there exists a correlation between caloric restriction and life span extension (Figure 3) (Sundaresan et al., 2012). Many past studies established that one of the benefits of CR diets is the decrease in electron leakage and prevention of membrane potential loss within mitochondria (López-Lluch et al., 2006). Senescent mice given a calorie-restricted diet have been observed to maintain metabolic dependence on β-oxidation, and thus reduced the tendency of the old heart to switch substrates from fats to glucose, compared to age-matched mice on a typical diet (Dai et al., 2014). Another investigation yielded evidence that suggests that a 40% caloric reduction prevented a maladaptive redox state shift of reduced to oxidized glutathione (GSH to GSSG) (Rebrin et al., 2003). Interestingly, CR also correlates with the reduced expression of pro-apoptotic genes such as Bax, Bad, Caspase 9, and Capsase 11, whereas CR upregulated the expression of anti-apoptotic regulator Bcl-x (Lee et al., 2002), and can also induce mitochondrial biogenesis as well as bioenergetic efficiency (López-Lluch et al., 2006). While earlier studies have shown that CR can promote mitochondrial biogenesis in healthy and young human skeletal muscle (Nisoli et al., 2005; Civitarese et al., 2007), other reports have observed no connection between CR and mitochondrial biogenesis within the mammalian heart (Hancock et al., 2011). One group of investigators have postulated that restricting caloric intake without inducing malnutrition can lower mitochondrial free radical production as a therapeutic avenue for attenuating oxidative stress in the muscles of healthy humans, which may pose a translational solution for aging cardiac tissue (Civitarese et al., 2007). Finally, several reports demonstrate that CR can lower the chances of developing CVD and contractile dysfunction, observed in both murine and human models (Fontana et al., 2004; Wagh and Stone, 2004; Meyer et al., 2006; Seymour et al., 2006) and short-term CR suppress gene expression of key regulators involved with cardiac fibrosis (Dhahbi et al., 2006).

Another form of dietary restriction that positively effects health throughout aging is intermittent fasting (IF). Alternate-day fasting is a form of IF that has been attributed to beneficially lowering hypertrophic phenotypes in aged rat hearts by reducing age-induced inflammation by inhibiting oxidative damage (Figure 3) (Castello et al., 2010). In addition to ameliorating hypertrophy, alternate-day fasting also extended life-span and rescues homeostatic metabolism in humans and rhesus monkey subjects (Anderson et al., 2009; Colman et al., 2009). Researchers suggest that the mechanism of action behind IF is downregulation of pathological myocardial hypertrophic markers such as extracellular signal-regulated kinase 1 and 2 (ERK1/2) and phosphoinositide 3-kinase γ (PI3Kγ) (Castello et al., 2011). It has become clear that regulating the diet can impose promising health benefits for extending human life by delaying the onset of a normal aging phenotype.

Since oxygen radicals and other reactive molecular species have been heavily implicated in the progression of degenerative diseases, antioxidant supplements have become more commercially advertised and sold under the societal premise that they improve health (Gutteridge and Halliwell, 2010). Antioxidant-rich diets have also been considered for a series of clinical trials with the goal of minimizing the elevated levels of oxidative stress that correlates with age. Unfortunately, many of these trials and experiments did not observe a positive effect with increasing dietary antioxidant supplements with regard to onset prevention or reducing symptoms of age-related diseases (Figure 3) (Sadowska-Bartosz and Bartosz, 2014; Conti et al., 2016; Gutteridge and Halliwell, 2018). In fact, administering large doses of antioxidant not only has demonstrated in many cases no therapeutic effect, but it has also been associated with worsening pathological outcomes in human subjects. This unexpected finding has been termed “the antioxidant paradox” since this outcome has puzzled investigators (Halliwell, 2000). Instead of supplying high concentrations of antioxidants in the diet, it seems that a more effective approach may be to promote and stimulate endogenous antioxidant defenses. An aforementioned study observed that overexpression of mitochondrial targeted catalase in transgenic rats extend life by increasing antioxidant defenses, therefore there may be promise in increasing intracellular mitochondrial antioxidants instead of obtaining them externally (Schriner et al., 2005; Dutta et al., 2014). Additionally, investigations combining antioxidant rich diets with exercise regimens may show an additive effect since some studies demonstrate increasing exercise also increases oxidative stress.

Polyphenols, which are currently on the market as dietary antioxidants, can be detected at the level of the murine heart after digestion (Manach et al., 2004). A recent investigation determined that consumption of traditional Chinese medicine (TMC), which contains a variety of polyphenol antioxidants, may serve as a powerful intervention against cardiac aging phenotypes by means of targeting the UPS (Chen et al., 2021). One of the most popular dietary antioxidant polyphenol compounds is resveratrol, which was demonstrated to have anti-aging and cardioprotective effects through a nitric oxide (NO)-dependent mechanism (Figure 3) (Das et al., 2011). One recent study has shown that old rats supplemented with resveratrol over a 10-week period exhibited significantly reduced cardiac inflammatory, oxidative, and apoptotic markers (Torregrosa-Muñumer et al., 2021). Previous evidence suggests that resveratrol can also reverse cardiac hypertrophy and contractile dysfunction after it develops (Juric et al., 2007; Chan V. et al., 2011; Thandapilly et al., 2013). Pre-clinical studies demonstrated that resveratrol was promising in treating CVD in mice models (Zordoky et al., 2015), which led to testing its translation to humans. Several randomized clinical trials performed over the span of a decade provided evidence that when resveratrol is administered to human patients with heart failure, there were improvements to left ventricle diastolic function (Magyar et al., 2012; Militaru et al., 2013; Dyck et al., 2019). However, some resveratrol-focused clinical studies produced contradicting and insignificant results (Smoliga et al., 2013). Furthermore, there exists no consensus in the mechanism of resveratrol, as it has been hypothesized to metabolically stimulates antioxidant activity to mobilize against oxidative stress (Carrizzo et al., 2013), activates deacytelase SIRT1 for the induction of mitochondrial biogenesis (Arunachalam et al., 2010), and/or induce metabolic sensor AMPK activity to accomplish beneficial vasodilatory effects (Dolinsky et al., 2013). Additional studies are needed for resveratrol to be considered a reliable treatment against the age-related decline of heart function in humans.

As previously discussed, the sirtuin proteins have been recognized as therapeutic candidates for enhancing the longevity of heart health with respect to aging. Interestingly, caloric restriction has been shown to activate sirtuin 6 (SIRT6) to promote longevity and anti-aging in aged Fisher 344 rats (Kanfi et al., 2012). Due to the beneficial anti-aging potential of the sirtuin family, pharmacological SIRT-activators have been clinically implemented. Thus far, the effects of SIRT1-activating compound SRT1720 have resulted in an enhancement of cardiac contractility in aged mice hearts (Figure 3) (Ren et al., 2017). Administration of polyamine spermidine, another hypothesized stimulator of SIRT1, to old C57BL6 mice remediated aging phenotypes such as hypertrophy and systolic/diastolic pump function (Eisenberg et al., 2016). Although these compounds have yet to be tested in human aging studies, this evidence has certainly garnered attention and may have therapeutic potential (Wirth et al., 2019).

To summarize the main findings regarding caloric restriction, intermittent fasting, and exercise with regard to the aging heart as it currently stands, there is increasing evidence that restricting caloric intake or increasing energy expenditure through physical endurance training is beneficial for the heart. In regards to mitochondrial quality control and turnover, it is speculated that reducing calories leads to increased autophagy and probably mitophagy which in turn may enhance mitochondrial fitness. These models demonstrate the need to expand our understanding about health and diet, and the diets discussed within this review should be further evaluated especially for older aged individuals.

## 9 Future perspectives

The American Heart Association has reported that despite advancements for improving cardiac health, the prevalence of CVD is still escalating in the elderly population (Benjamin et al., 2019). Targeting the factors responsible for the harmful and often fatal deterioration of the heart has remained elusive, partly due to current ineffective treatments, as well as the complexity of age-related CVD as a whole. Here we have highlighted the evidence reported within the literature concerning the multifaceted forms of dysfunction and dysregulation that altogether amalgamate into the downfall of the aging myocardium. According to the general consensus, the contributors of age-related myocardial dysfunction compromise maladaptive changes in metabolism, dysregulation of redox reactions, poor protein quality control, decreased mitochondrial biogenesis, and induction of pro-cell death pathways (Paneni et al., 2017), all of which lead to increased cardiac dysfunction. Despite the collection of knowledge we have gained over the last several decades, there is still much we do not understand about cardiac aging.

Some questions indeed remain, for example; at the onset of cardiac-aging phenotypes, which signaling pathways are the primary drivers cardiac aging? Can these pathways be targeted in combination for additive protection? What is the maximal capacity of the human lifespan if optimal heart health is maintained? Once these questions are answered, we may eventually be able to attenuate negative aging defects. Notably, few current methods reduce maladaptive myocardial remodeling in humans in late adulthood. While changes in lifestyle such as exercise and diet are the most powerful behavioral interventions to thwart cardiac dysfunction, we must determine additional ways to minimize the associated side effects such as oxidative stress and potential nutrition deficit.

As we progress into the future, the goal of maintaining cardiac health during aging will become more feasible with the identification of novel therapeutic targets. The objective of therapeutic interventions should be to target cell death, ensure long-term mitochondrial metabolic function, and prevent the accumulation of toxic or dysregulated cellular components. Treatments that address more global, multi-faceted aspects of cardiac aging will ensure long-term mitochondrial health within the elderly heart. With these combined efforts, it is just a matter of “time” until we reap the benefits of maintaining heart health into old age and expand the boundaries of human life.
